# A Thermo-Photo-Ionic Crosslinked Gellan Gum Hydrogel with Gradient Biomechanic Modulation as a Neuromaterial for Peripheral Nerve Injury

**DOI:** 10.3390/gels11090720

**Published:** 2025-09-10

**Authors:** Sameera Khatib, Poornima Ramburrun, Yahya E. Choonara

**Affiliations:** Wits Advanced Drug Delivery Platform Research Unit, Department of Pharmacy and Pharmacology, School of Therapeutic Sciences, Faculty of Health Sciences, University of the Witwatersrand, Johannesburg 2193, South Africapoornima.ramburrun@wits.ac.za (P.R.)

**Keywords:** gellan gum, hydrogels, plasticizer-modified hydrogels, photo-crosslinking, ionic crosslinking, thermal gelation, hydrogel characterization, peripheral nerve regeneration

## Abstract

Gellan gum (GG) is a promising biomaterial due to its biocompatibility, tunable gelation, and modifiability. This study investigates the influence of triple crosslinking mechanisms—thermal gelation, UV-induced covalent crosslinking, and ionic crosslinking—on the mechanical and physicochemical properties of GG-based hydrogels, designed to function as a neuromaterial with hierarchical neuro-architecture as a potential nerve substitute for peripheral nerve injury. Initial thermal gelation forms a physical network via double-helix junctions. Methacrylation introduces vinyl groups enabling UV crosslinking, while post-treatment with Mg^2+^ ions strengthens the network through ionic bridging with carboxylate groups. Plasticizers—glycerol and triethyl citrate—were incorporated to modulate chain mobility, network hydration, swelling behavior, and mechanical flexibility. Seven-day erosion studies showed that glycerol-containing hydrogels eroded 50–60% faster than those with triethyl citrate and up to 70% more than hydrogels without plasticizers, indicating increased hydrophilicity and matrix loosening. In contrast, triethyl citrate reduced erosion, likely due to tighter polymer chain interactions and reduced network porosity. Mechanical testing of 1% *v*/*v* methacrylated GG hydrogels revealed that 1.5% *v*/*v* triethyl citrate combined with UV curing (30–45 min) produced tensile strengths of 8.76–10.84 MPa. These findings underscore the synergistic effect of sequential crosslinking and plasticizer choice in tuning hydrogel mechanical properties for neuro application. The resulting hydrogels offer potential as a neuromaterial in peripheral nerve injury where gradient mechanical properties with hydration-responsive behavior are required.

## 1. Introduction

Peripheral nerve injuries (PNI) occur due to partial or complete separation of a peripheral nerve, resulting from traumatic injury, laceration wounds, or crushing or stretching injuries [1]. Surgical treatments are necessary for functional recovery, and the current gold standard, autographing, poses issues such as donor-injury site mismatch and donor site morbidity [2]. Nerve guidance conduits (NGCs) have emerged as a mainstay of treatment for PNIs when autologous nerve transplants are non-viable. Traditional NGCs lacking biomimetic structures have been associated with prolonged recovery times and reduced functional recovery [3]. While FDA-approved conduits have shown promising results, the need for NGCs with superior outcomes remains, as currently available NGCs do not provide the injured nerve with an ideal microenvironment for regeneration [4,5].

Biomimetic NGCs provide an ideal solution. Biomimetic biodegradable NGCs have been shown to improve nerve morphology and function, axonal growth, and myelin tissue formation [6,7], as well as eliminating the need for a second surgical procedure [8]. These advantages rely on replicating multiple intricacies of the native design of peripheral nerves. Biomaterial selection, structural designs, and manufacturing techniques all play a vital role in effective biomimetic NGCs, impacting biocompatibility, degradation rate, and mechanical properties [9].

Natural polymers are preferable due to increased biocompatibility and biodegradability; however, they are limited by poor mechanical strength, such as gellan gum (GG) [10,11]. The hierarchical structural design of peripheral nerves, specifically the epineurium, perineurium, and endoneurium, provides an ideal guide for mimicking biomechanical properties necessary for nerve regeneration [12].

Techniques such as solvent casting are advantageous in their simplicity: they provide a structural advantage, allowing for the formation of porous structures [13], remain cost-effective due to minimal use of highly specialized equipment [14], and vitally provide versatility in material selection, enhancing biocompatibility and biodegradability through natural polymer use [15].

The aim of this study was to create a GG scaffold using a triple crosslinking approach and investigate plasticizer selection for tunable mechanical properties to best mimic native nerve tissue textural properties of the peripheral nervous system using the solvent casting method to develop a neuromaterial for peripheral nerve injury.

## 2. Results and Discussion

### 2.1. Results

#### 2.1.1. Confirmation of Chemical Transitions Using Fourier Transform Infrared Spectroscopy (FTIR)

To confirm the chemical structures and detect molecular transitions thereof, samples of the conduits were analyzed for the presence of crosslinking and interactions between the native polymers gellan gum (GG) and methacrylic acid (MAA). Compositions for formula codes are listed in Table 1.

**Figure 1:**
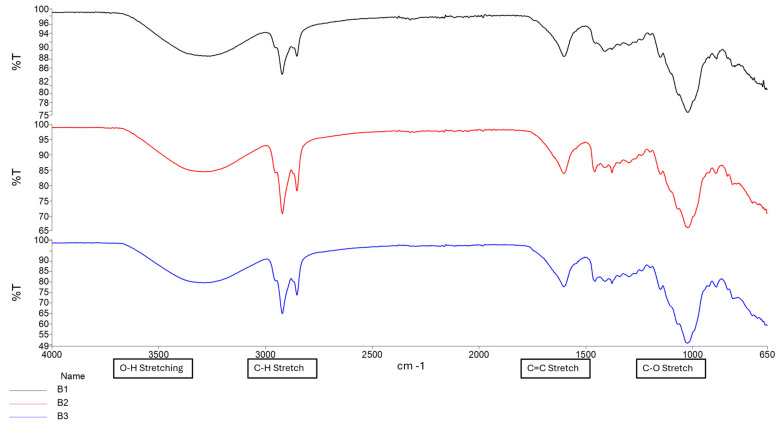
FTIR spectrum of Samples B1–B3.

The ionic crosslinking of GG with MgCl_2_ results in distinct FTIR peaks that indicate complex formation and structural changes as depicted in Figure 1. Broad O-H stretching at 3200–3600 cm^−1^ indicates the presence of hydroxyl groups essential for the formation of hydrogen bonds in the GG structure. The shift observed in conduit samples between 1000–1200 cm^−1^ (C-O Stretching) indicates changes in the GG polymer backbone due to the incorporation of magnesium ions. This differs from the C-O bond observed in samples B1–B3 due to the alcohol C-O stretch that appears strong between ~1040–1100 cm^−1^, which is present in Glycerol but not Tri-ethyl citrate [16]. The peak observed around 2850–2950 cm^−1^ (C-H stretching) indicates the presence of ethyl groups from the plasticizer triethyl citrate (TEC) and decreases in intensity as plasticizer concentration increases.

**Figure 2:**
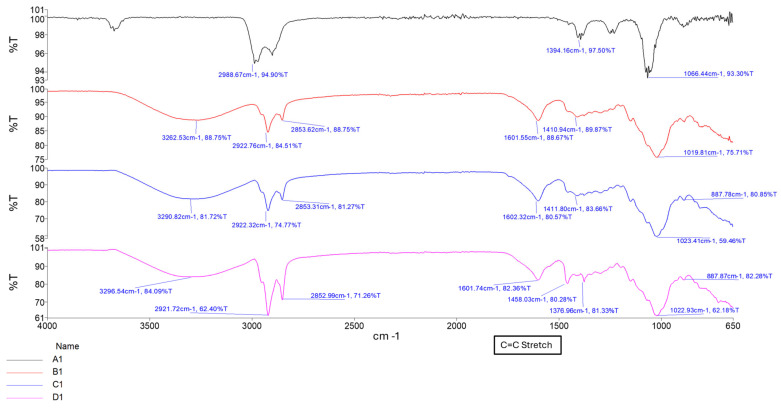
FTIR spectrum of samples A1, B1, C1, and D1, showing increasing curing time.

**Figure 3:**
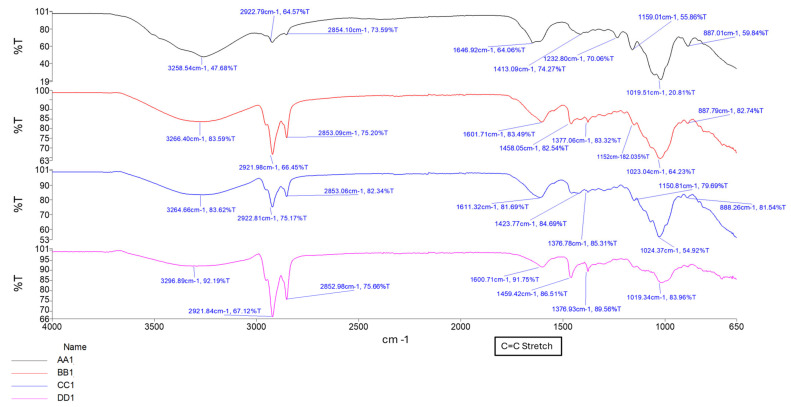
FTIR spectrum showing samples AA1, BB1, CC1, and DD1, containing glycerol at different curing times.

**Figure 4:**
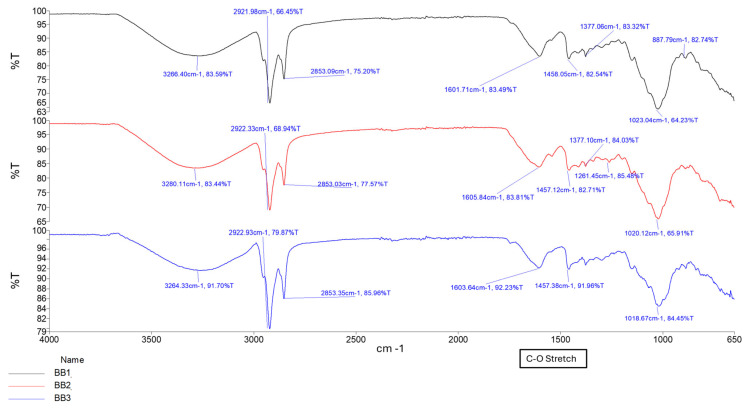
FTIR spectrum of samples BB1, BB2, and BB3, showing different glycerol concentrations.

The formation of a C=C stretching between 1630 cm^−1^ and 1640 cm^−1^ was observed as the increased intensity of the peak, along with changes in the carboxylate groups near 1720 cm^−1^, confirming successful methacrylation of GG polymer and formation of a hydrogel matrix. The intensity of the peak between 1630 cm^−1^ and 1640 cm^−1^ increases with increased crosslinking time, as depicted in Figure 2 and Figure 3 [16,17]. Figure 4 depicts the C-O stretching vibrations in glycerol observed around 1045–1080 cm^−1^, with increased intensity as glycerol concentration increased [18].

#### 2.1.2. Swelling and Erosion Analysis

**Figure 5:**
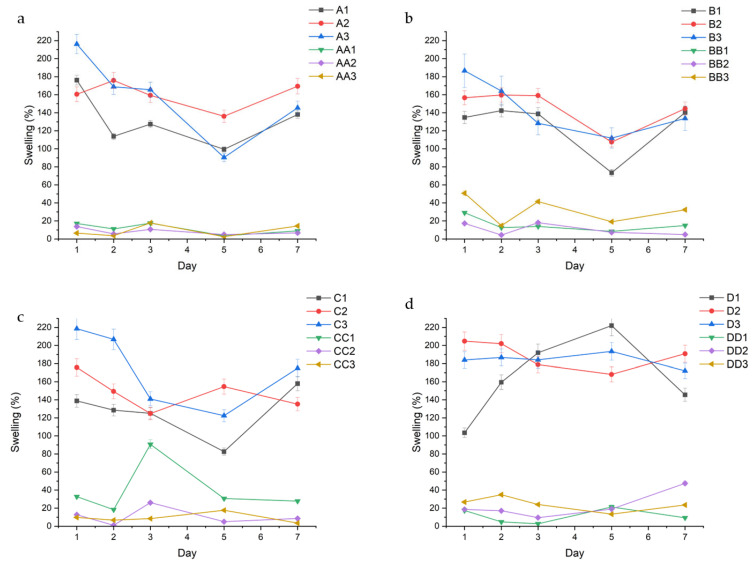
Swelling % of different conduits over time, grouped in accordance with curing time; (**a**) 10 min, (**b**) 20 min, (**c**) 30 min, and (**d**) 45 min.

Swelling studies were conducted to evaluate the fluid absorption behavior and swelling characteristics of the conduits, starting from a dehydrated state, in response to different plasticizer concentrations and crosslinking durations. Figure 5 below shows the swelling behavior of the conduit samples in phosphate-buffered saline (PBS) containing biologically relevant salts at a pH of 7.4.

Triethyl citrate (TEC) containing samples consistently absorbed more fluid than glycerol samples. Lower plasticizer concentration resulted in the least fluid uptake, with minimal differences between median and higher concentrations, regardless of plasticizer type. For example, in Figure 5a, A1, A2, and A3, had an average swelling of 130.96% (±29.51), 160.21% (±15.12), and 157.29% (±45.5), while AA1, AA2, and AA3 had an average swelling of 11.66% (±5.9), 8.37% (±3.76), and 9.0% (±6.79), respectively. Samples containing median plasticizer concentration (1.5% *v*/*v*) maintained the most consistent swelling profiles at lower curing times (10–20 min) as evidenced by their standard deviation. For example, samples B1 and BB1 demonstrated swelling ratios of 125.92% (±29.44) and 15.81% (±7.86), while samples B2 and BB2 had swelling ratios of 145.48% (±22.09) and 10.41% (±6.74), respectively (Figure 5b). At higher curing times (30–45 min), samples with higher plasticizer concentration (2.0% *v*/*v*) displayed lower swelling variability. This is highlighted in the D and DD series, with D1 and DD1 having an average swelling of 164.48% (±45.20) and 11.12% (±8.03), compared to D2 and DD2 with an average swelling of 188.93 (±15.53) and 22.35% (±14.57), respectively.

**Figure 6:**
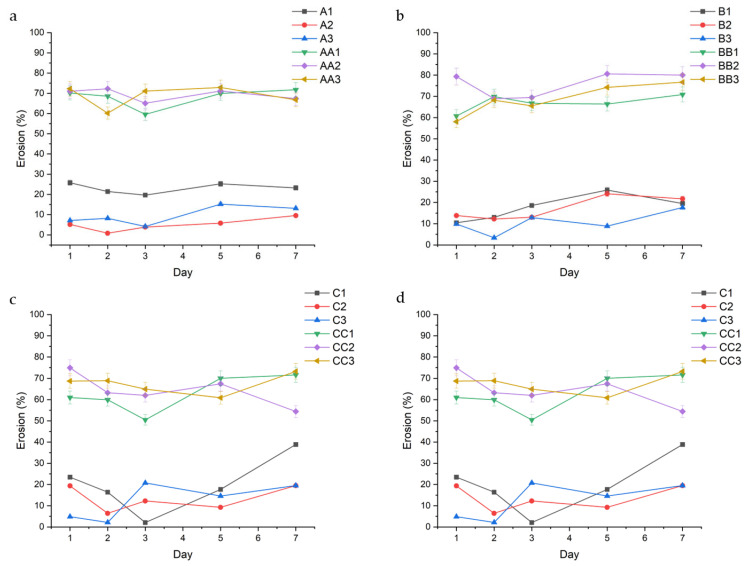
Erosion behavior over 7 days of the hydrated conduits, grouped in accordance with curing time. Series A ((**a**)—10 min), Series B ((**b**)—20 min), Series C ((**c**)—30 min), and Series D ((**d**)—45 min) are highlighted above.

Erosion analysis was performed to assess the impact of fluid uptake on the structural stability and degradation behavior of the hydrogel conduits over time. Figure 6 describes the erosion profiles of the conduits. Erosion rates displayed the opposite trend to swelling, wherein glycerol conduits eroded at a consistently higher rate than TEC-containing conduits.

The data show a direct correlation between curing time and erosion, with conduits cured for 45 min displaying the lowest rates of erosion. This can be observed across conduits containing 1.0% TEC, with samples A1, B1, C1, and D1 displaying a decreasing average erosion rate of 23.06% (±2.56), 17.49% (±6.02), 19.70% (±13.30), and 6.81% (±3.60), respectively (Figure 2). In samples containing glycerol, the erosion rate is consistent regardless of curing time, with samples AA1, BB1, CC1, and DD1 displaying a somewhat consistent erosion rate of 67.99% (±4.82), 66.91% (±3.99), 62.61% (±8.57), and 69.71% (±7.82), respectively. TEC concentration played a role in erosion rate, with 1.5% *v*/*v* TEC resulting in decreased erosion. This is observed in the A-series in Figure 6a, with samples A1, A2, and A3 having an erosion rate of 23.06% (±2.56), 5.01% (±3.17), and 9.55% (±4.51), respectively. A similar trend is observed across the B, C, and D series.

#### 2.1.3. Mechanical Performance Metrics

##### Matrix Resilience (MR)

MR quantifies the elastic efficiency of the hydrogel and reflects its capacity to store and release mechanical energy without undergoing permanent deformation [19].

**Figure 7:**
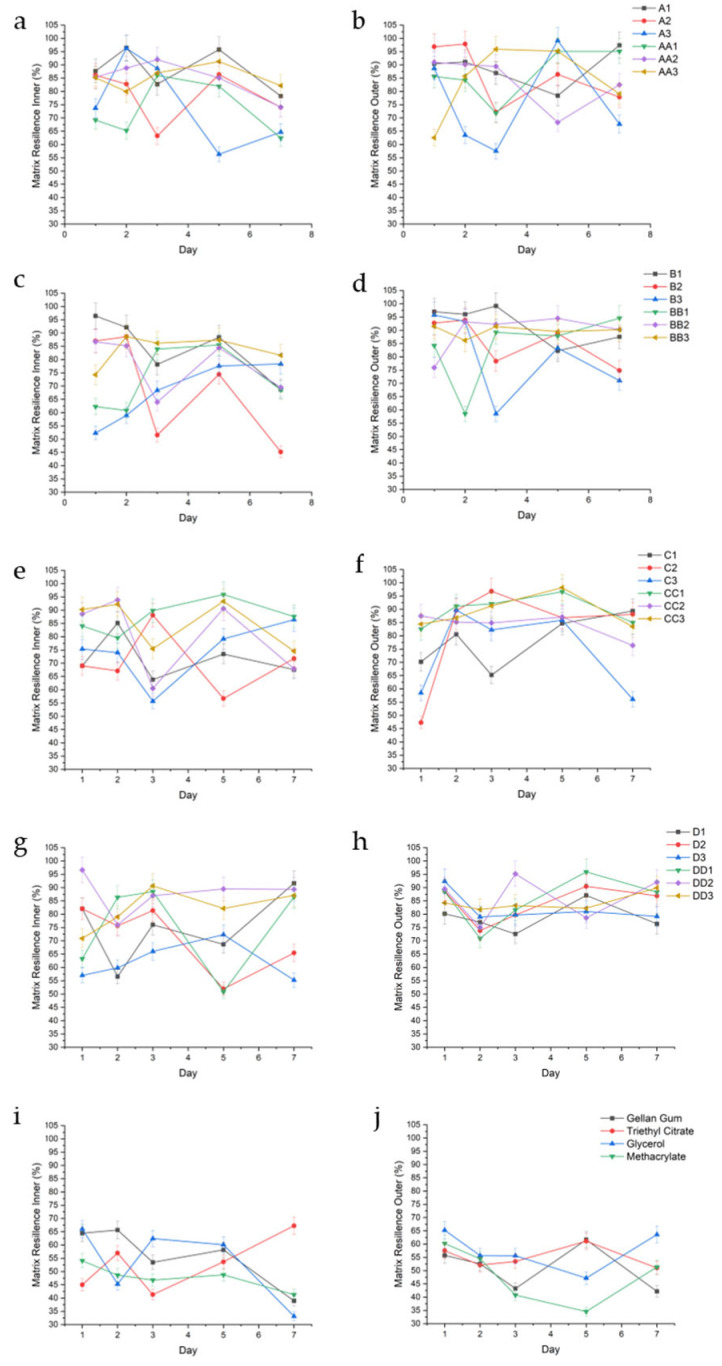
Graphical representation of %MR of the inner and outer regions of conduit samples cured for 10 min (**a**,**b**), 20 min (**c**,**d**), 30 min (**e**,**f**), 45 min (**g**,**h**), and controls (**i**,**j**).

MR values exhibited distinct differences between the inner and outer layers of the hydrogel conduits. Outer matrix regions consistently demonstrated higher resilience compared to their inner counterparts across most formulations and time points. For example, the outer MR values of the A-series depicted in Figure 7a were 88.88% (±6.95), 86.24% (±11.34), 75.32 (±17.77), 86.39 (±9.61), 84.28% (±9.54), and 83.69% (±13.73) for samples A1, A2, A3, AA1, AA2, and AA3, respectively. The corresponding inner MR values for those samples, shown in Figure 7b, either decreased or remained the same at 88.16% (±7.97), 78.50% (±9.90), 75.91% (±16.52), 72.95% (±10.45), 85.06% (±6.77), and 85.11% (±4.38).

With conduits containing triethyl citrate as a plasticizer, samples cured for 10 and 20 min showed increases in swelling, resulting in decreased matrix resilience. For example, sample A1 had lower inner MR values on days 1 and 3 of 81.69% (±9.12) and 82.73% (±2.66), respectively, corresponding with higher swelling of 176.16% and 127.38%. In the same sample, when swelling decreased to 113.83% and 99.35% on days 2 and 5 (Figure 5a), MR increased to 96.40% (±3.69) and 95.76% (±3.17). This trend is observed in the inner portion of samples B1 (on days 2 and 3), A2 (on day 7), and B2 (on days 3 and 7). In samples cured for 30 to 45 min, the trend is reversed, where increased matrix resilience correlated with increased swelling. This is first observed on the inner portions of conduits C1 on day 7, D1 on days 3 and 5, and D2 on day 7. This trend is highlighted on the inner section of conduits containing 2.0% triethyl citrate, wherein B3 (20 min UV crosslinking) shows an increased matrix resilience trend with decreased swelling, while D3 (45 min UV crosslinking) shows an increased matrix resilience trend with increased swelling. The outer portion of the conduits demonstrated a similar trend, where sample D1 increased in MR with increased swelling (day 5), and samples C2, D2, and D3 showed consistently high MR with consistently high swelling. The outer portions of conduits cured between 10 and 20 min showed a similar trend (A3, day 2–7; B3, day 5). All samples displayed a decrease in MR over time, attributed to erosion.

Samples containing 1.0% glycerol (*v*/*v*) behaved unpredictably regardless of curing time. For example, sample AA1 exhibited variations in inner MR despite relatively consistent swelling with an average swelling of 11.66% (±5.9), while BB1 displayed a decline in inner MR after day 3 without a significant change in swelling. Higher curing times (CC1—30 min) resulted in consistently high inner MR with consistent swelling. However, this effect was lost beyond 30 min curing time. As glycerol concentrations increased (1.5% and 2.0% *v*/*v*), inner and outer portions of the conduit had lower MR when swelling increased, regardless of curing time, observed across all samples in the groups.

##### Deformability Modulus (DM)

DM was used to assess the matrix stiffness of the conduits, which is especially vital for applications requiring mechanical compatibility with soft biological tissues.

**Figure 8:**
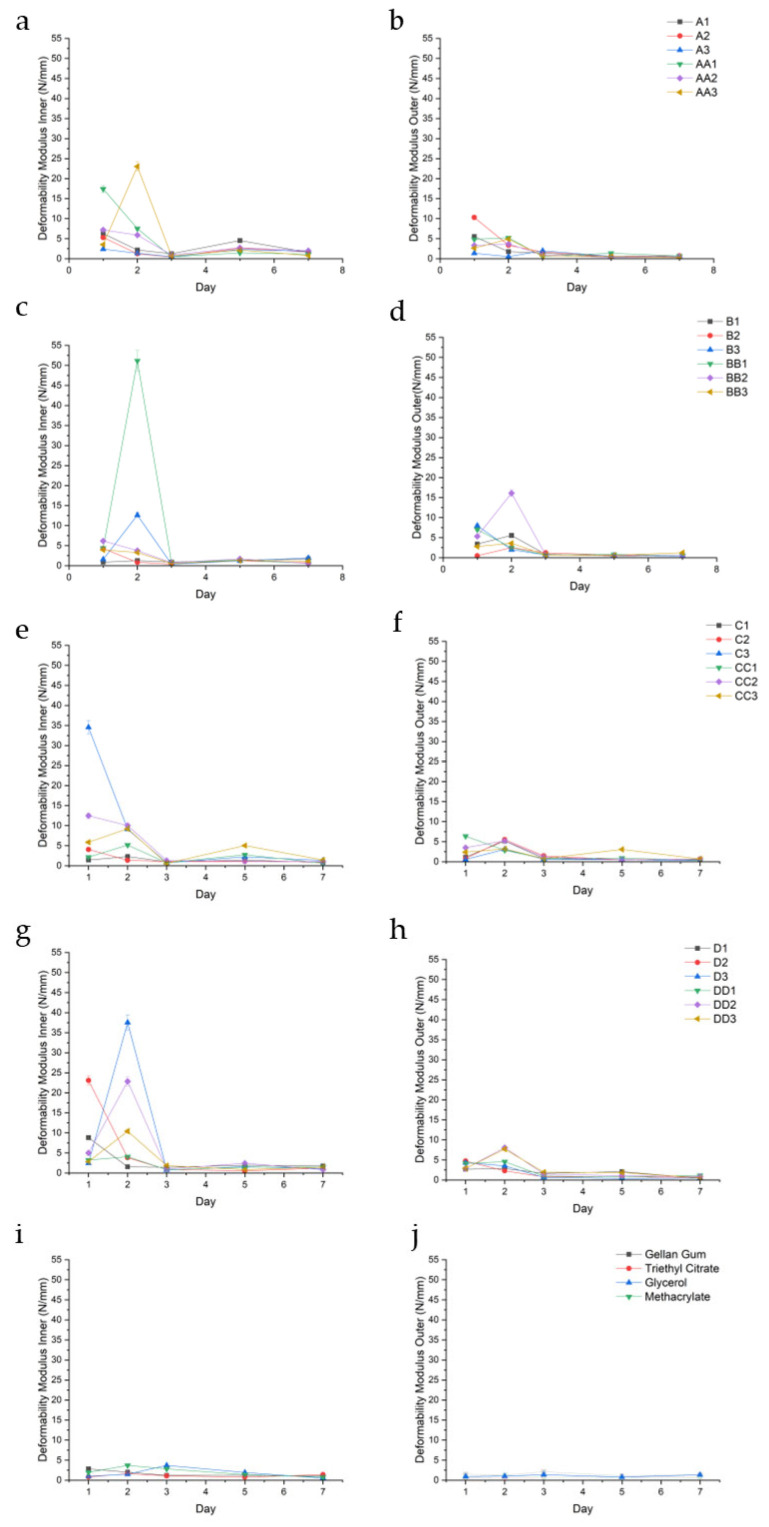
Deformability modulus (N/mm) of the inner and outer regions of conduit samples cured for 10 min (**a**,**b**), 20 min (**c**,**d**), 30 min (**e**,**f**), 45 min (**g**,**h**), and controls (**i**,**j**).

DM values showed a consistent and measurable distinction between the inner and outer regions of the hydrogel conduits, reflecting a radial gradient in mechanical flexibility, dependent on UV curing time. Samples cured for 10 min displayed a higher inner DM compared to outer, as observed in samples A1, A3, AA1, AA2, and AA3. The inner DM values for these samples, respectively, depicted in Figure 8a, were 3.16 (±2.17), 1.67 (±0.79), 5.60 (±7.20), 3.74 (±2.67), and 6.04 (±9.57) and were higher than the outer values of 1.95 (±2.08), 0.98 (±0.66), 2.54 (±2.30), 1.75 (±1.64), and 1.83 (±1.96) as depicted in Figure 8b.

This phenomenon was independent of curing time, exemplified by the D-series, wherein the average outer DM values of D1, D2, D3, DD2, and DD3 were 1.94 (±0.98), 1.86 (±1.74), 1.88 (±1.89), 2.87 (±3.01), and 3.00 (±2.76), respectively, while the inner DM values were consistently higher at 3.10 (±3.21), 5.91 (±9.70), 8.69 (±16.14), 6.43 (±9.34), and 3.48 (±3.95). Sample DD1 was a notable exception with outer and inner DM values of 2.32 (±1.88) and 2.21 (±1.36). All values were reported in N/mm.

The outer hydrogel layers typically exhibited lower deformability modulus values compared to the inner layers, indicating greater compliance and a higher capacity to undergo elastic deformation before reaching peak stress. In contrast, the inner layers demonstrated relatively higher deformability modulus values, denoting a stiffer, less extensible network.

DM consistently peaks with increased swelling in samples containing TEC at all concentrations; for example, sample B1 experienced a peak in DM on day 2 (5.57 N/mm ±0.29), corresponding with a higher swelling of 142.33%. Some samples demonstrated a decreased DM with reduced swelling, such as the outer portion of conduit D2 observed over days 1–3. Glycerol-containing samples demonstrated the opposite effect, wherein increased swelling resulted in a consistently reduced DM. Sample BB2 (Figure 8c) displays a high DM inner on day 1 of 6.17 N/mm (±0.48), corresponding to a high swelling of 29.18%, depicted in Figure 5b. However, glycerol-containing conduits generally had a higher DM compared to TEC conduits. Samples containing TEC yielded stiffer, less deformable matrices, particularly at higher concentrations and longer curing times. Both TEC and glycerol improved DM when compared to controls, with the combination of plasticizer and UV cross-linking producing a profound improvement in the DM of all sample sets.

##### Fracture Energy (FE)

**Figure 9:**
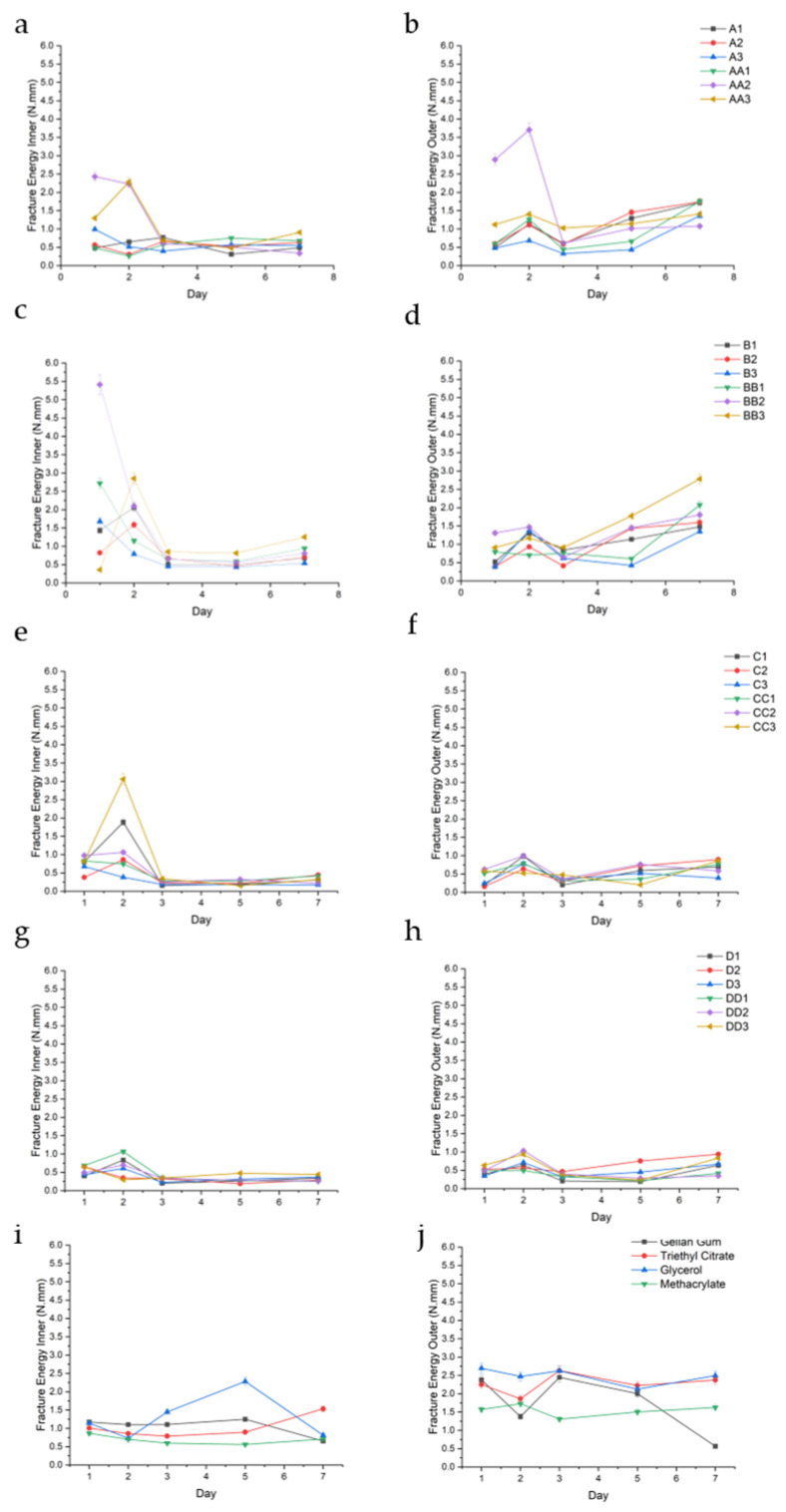
FE (N.mm) of the inner and outer regions of conduit samples cured for 10 min (**a**,**b**), 20 min (**c**,**d**), 30 min (**e**,**f**), 45 min (**g**,**h**), and controls (**i**,**j**).

FE was assessed to quantify the hydrogels’ toughness and ability to withstand compressive forces before rupturing or deforming. Figure 9 below depicts the FE for the conduit samples over a 7-day period.

FE analysis revealed a consistent and measurable gradient between the inner and outer matrix regions of the hydrogel conduits across all formulations and time points. The inner matrix generally exhibited significantly lower fracture energy values compared to the outer matrix, regardless of curing time or plasticizer composition. This spatial trend was observed uniformly, indicating a robust difference in mechanical toughness along the radial axis of the conduits. This is best observed in the A-series, wherein outer FE for samples A1, A2, A3, AA1, AA2, and AA3 were 1.04 (±0.51), 1.10 (±0.51), 0.66 (±0.41), 0.94 (±0.55), 1.86 (±1.360), and 1.22 (±0.18), while the corresponding inner FE was consistently lower at 0.54 (±0.17), 0.63 (±2.2), 0.60 (±0.23), 0.55 (±0.19), 1.22 (±1.02), and 1.13 (±0.71) (Figure 9a,b), respectively.

Among the tested samples, BB2, C3, and CC3 exhibited the highest inner matrix fracture energy values, with BB2 (1.5% glycerol, 20 min UV) reaching up to 5.41 N·mm (Figure 9c,e,f). This corresponded to a high swelling observed at the same time point. This trend was observed throughout the samples. These samples demonstrated a favorable combination of moderate outer FE values with elevated inner toughness, indicating a strong internal structural profile. Across most formulations, fracture energy values peaked on day 2, particularly within the inner matrix, followed by either stabilization or gradual decline in subsequent time points.

##### Tensile Strength (TS)

TS testing was conducted to assess the mechanical robustness of the hydrogel conduits under uniaxial stress.

**Figure 10:**
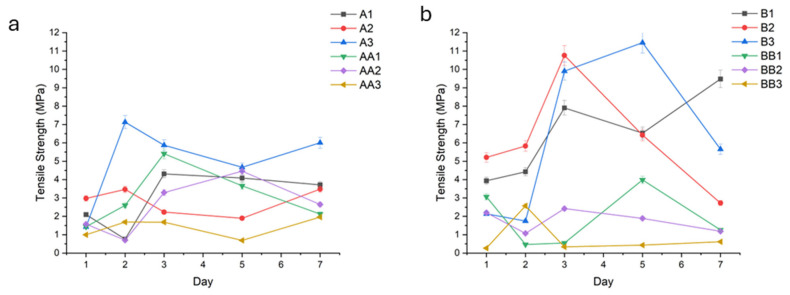

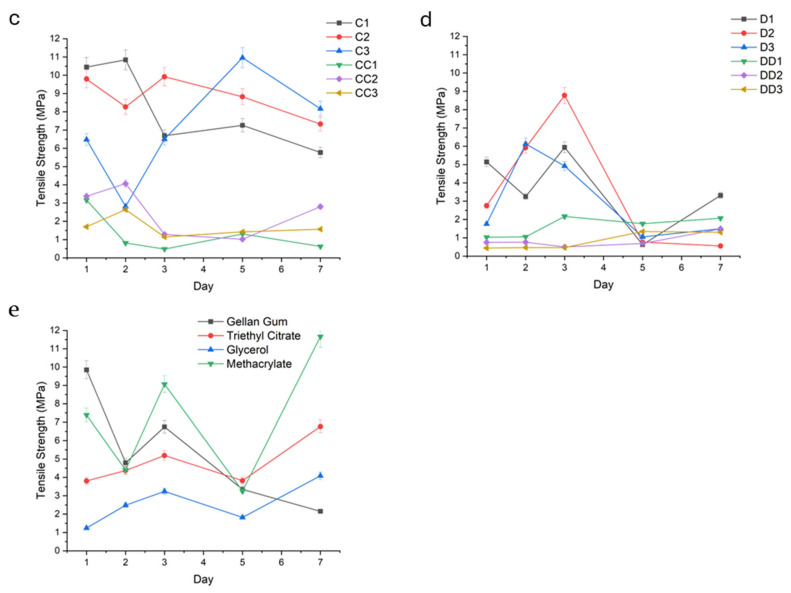
Fracture energy (N.mm) of the conduit samples cured for 10 min (**a**), 20 min (**b**), 30 min (**c**), 45 min (**d**), and controls (**e**).

An increase in TS correlated positively with prolonged UV curing times, ranging from 10 to 30 min. Samples exposed to 30 min of UV light exhibited significantly higher tensile strength compared to those cured for 10–20 and 45 min (A, B, and D series). For example, samples A1, B1, and D1 (Figure 10a,b,d) demonstrated tensile strengths of 3.00 MPa (±1.52), 6.45 MPa (±2.33), and 3.65 MPa (±2.05), while sample C1 (Figure 10c) had a TS of 8.20 MPa (±2.30), respectively.

Hydrogels plasticized with TEC (e.g.,A1, A2, A3, B1, B2, B3, C1, C2) demonstrated superior tensile strength in the upper range of 6.00 MPa (±1.07) to 10.45 MPa (±0.52), especially at low (1.0%) and intermediate (1.5%) concentrations. In contrast, glycerol-plasticized hydrogels (i.e., AA1-3, BB1-3, CC1-3) exhibited lower tensile strengths, particularly at elevated concentrations in the upper range of 3.66 MPa (±0.97) to 5.41 MPa (±1.31).

Such improvement observed with increased curing time was not apparent in conduits containing glycerol.

##### Young’s Modulus

Modulus values were calculated to determine the conduits resistance to stretch in the elastic range.

**Figure 11:**
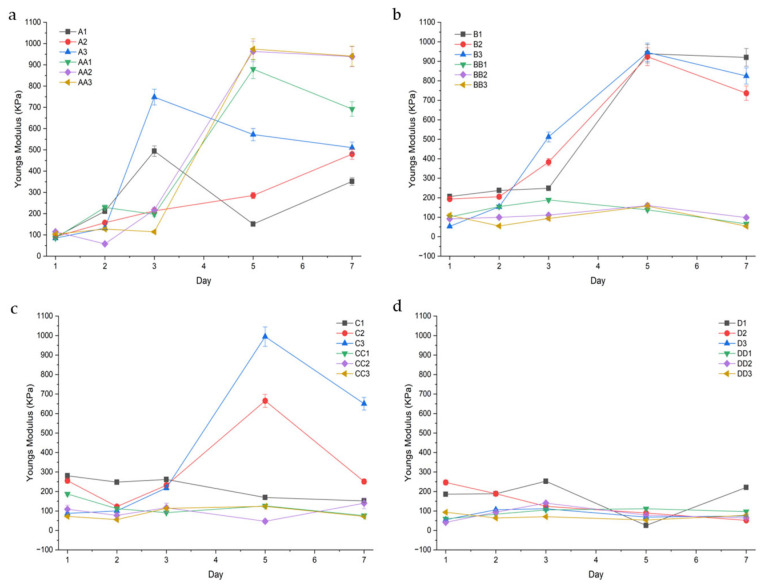
Youngs modulus (KPa) of the conduit samples cured for 10 min (**a**), 20 min (**b**), 30 min (**c**), and 45 min (**d**).

Samples that were cured for 10 min and contained glycerol had higher and more varying modulus values, depicted above in Figure 11. For example, on Day 5, samples AA1 (10 min, 1%GLY), AA2, and AA3 displayed a modulus of 879.57 Kpa (± 138.44), 962.88 (±39.90), and 974.20 (±21.37), respectively. Compared to their glycerol-containing counterparts, samples A1 (10 min 1%TEC), A2 (10 min 1.5% TEC), and A3 (10 min 2.0% TEC) were observed to have lower and more consistent modulus values of 151.03 (±43.88), 285.48 (±22.34), and 571.97 (±19.04), respectively. As curing time increased, samples were observed to have a lower modulus value. For example, samples BB1 (20 min 1%GLY), CC1 (30 min 1%GLY), and DD1 (45 min 1%GLY) displayed modulus values of 138.00 (±80.21), 126.00 (±36.17), and 111.33 (±43.82), respectively. TEC-containing samples displayed a higher and more variable modulus when exposed to shorter curing times, and a smaller, more consistent modulus when cured for 30 min or longer. Glycerol-containing samples displayed consistently lower modulus values when cured for 20 min or longer.

#### 2.1.4. In Vitro PC12 Proliferation

**Figure 12:**
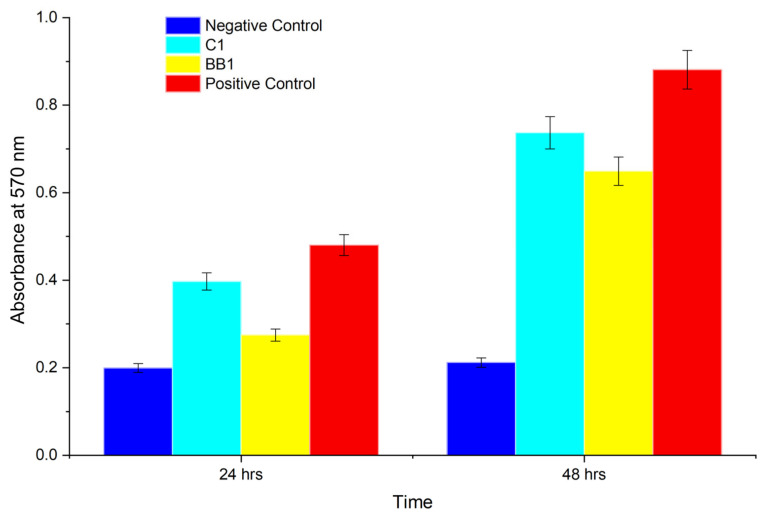
MTT viability assay of PC12 cells showing the negative control, sample C1 (1.5%TEC 30 min UV), sample BB1 (1.0% glycerol 20 min UV), and the positive control.

PC12 proliferation in response to selected conduits were determined over 24 and 48 h (Figure 12). Samples C1 (30 min 1.5%TEC) and BB1 (20 min, 1.0% GLY) were tested for cell viability both as a representative of each set of variables and due to their preferred mechanical properties comparable to those of native nerve tissue.

Samples C1 (1.5%TEC 30 min UV) supported cell growth comparable to the control, while sample BB1 (1.0% glycerol 20 min UV) had a lower viability (n = 3).

### 2.2. Discussion

#### 2.2.1. Triple Crosslinking Approach and Relevance to Native Nerve Tissue

This study employed a triple crosslinking strategy—thermal gelation, UV-induced photo-crosslinking, and ionic crosslinking—to synthesize GG hydrogels with biomimetic, tunable mechanical properties. Thermal gelation-initiated network formation via helical junction zones, UV exposure covalently fixed methacrylate groups into the polymer backbone, and Mg^2+^ ionic crosslinking introduced secondary electrostatic bridges with GG carboxylates on the outer surface of the conduits [12].

This sequential crosslinking mimics the multi-layered architecture of peripheral nerves, where the epineurium, perineurium, and endoneurium differ in stiffness and elasticity. For example, inner conduit regions consistently demonstrated higher DM and lower FE, while outer regions were stiffer and tougher, mirroring the gradient mechanical resilience observed in native nerve tissue [20]. This radial mechanical profile is particularly relevant for supporting axonal regrowth and preserving structural integrity during dynamic physiological loading.

#### 2.2.2. Effect of Plasticizer Selection and Concentration on Swelling, Erosion, and Mechanical Properties

Swelling behavior governs the diffusion of nutrients, waste, and signaling molecules through the hydrogel matrix, directly impacting cell viability and tissue integration. Swelling studies quantified the hydrogel’s fluid uptake capacity.

For nerve conduits, an optimal swelling ratio ensures hydration and flexibility without compromising mechanical integrity or dimensional stability. Excessive swelling may cause conduit deformation, lumen closure, or mechanical mismatch with surrounding tissues. Plasticizer incorporation dramatically influenced swelling, erosion, and mechanical performance of the hydrogel conduits.

Glycerol, a highly hydrophilic, low-molecular-weight triol, increased erosion due to its small size and ability to disrupt interchain hydrogen bonding, thereby loosening the network [21]. Conduits containing glycerol, i.e., AA (glycerol, 10 min UV) and BB (glycerol, 20 min UV) series, displayed high swelling variability and erosion rates upwards of 60–70%, independent of UV exposure duration. FTIR spectra confirmed glycerol’s presence by characteristic C–O stretching peaks between 1045–1080 cm^−1^, which intensified with increasing glycerol concentration. While Glycerol-containing hydrogels demonstrated a lower swelling ratio over the 7-day period, these samples eroded at a considerably higher rate. This increase in erosion corresponded with a reduction in tensile strength [22]. These effects are consistent with the known water-attracting and matrix-loosening properties of glycerol, which facilitate higher porosity and polymer diffusion into the medium [23].

In contrast, TEC-containing formulations exhibited reduced swelling and erosion, particularly at longer UV curing times. These samples maintained dimensional and mechanical stability over the test period, which can be attributed to their lower hydrophilicity and increased network rigidity [24]. TEC-containing samples consistently demonstrated lower swelling, reduced erosion, and significantly higher tensile strengths (up to 10.45 MPa), especially at 1.5% *v*/*v*. FTIR spectra confirmed TEC incorporation through characteristic C–H stretching peaks at 2850–2950 cm^−1^, which decreased in intensity as TEC concentration increased—suggesting effective plasticizer entrapment.

A direct correlation was observed between swelling behavior and mechanical degradation. Samples with high swelling ratios exhibited reduced fracture energy and tensile strength, particularly by day 3, indicating that prolonged exposure to aqueous environments compromises structural performance in highly hydrated networks. The erosion data further supported this conclusion, with the most erodible samples being those that also had the highest swelling and lowest deformability modulus values.

Deformability modulus (DM) refers to the effective modulus that characterizes a hydrogel’s ability to undergo elastic or plastic deformation prior to rupture. In TEC-containing conduits, DM increased consistently with swelling, especially in samples cured for longer durations and at higher plasticizer concentrations. This counterintuitive trend—where increased fluid uptake leads to increased stiffness—may be explained by the restricted mobility of the polymer chains at high TEC concentrations, where increased crystallinity limits matrix expansion [25]. Notably, the outer layer of D2 (1.5%TEC, 45 min UV) (Days 1–3) displayed the opposite, showing a decrease in DM with reduced swelling, possibly reflecting local heterogeneity in crosslinking or plasticizer diffusion.

Glycerol-containing samples demonstrated a more classical behavior, where swelling correlated with reduced DM. The hydrophilic and chain-mobility-promoting nature of glycerol facilitates fluid uptake, which in turn plasticizes the network, reducing stiffness and enhancing deformability. However, despite this, glycerol-containing conduits consistently exhibited higher DM values overall compared to their TEC counterparts. This may be attributed to glycerol’s dual role: while it softens the matrix, it also contributes to intermolecular cohesion through hydrogen bonding, leading to a less extensible but more uniformly compliant network [26].

FE is defined as the critical energy release rate required to propagate a pre-existing crack through the hydrogel matrix. It quantifies the material’s resistance to crack extension and is a measure of its intrinsic toughness. In hydrogels, particularly those intended for load-bearing or dynamic tissue engineering applications, high fracture energy is indicative of a well-integrated and dissipative polymer network capable of resisting mechanical failure under stress concentrations.

The FE values peaked around day 2, particularly in the inner regions, suggesting post-curing structural stabilization and possible network rearrangement driven by continued ionic interactions with Mg^2+^. This temporal maturation, coupled with swelling-induced stress equilibration, may explain the delayed maximization of fracture resistance. Notably, C3 (2.0% TEC, 30 min UV), BB2 (1.5% glycerol, 20 min UV), and CC3 (2.0% glycerol, 30 min UV) displayed the most mechanically resilient inner matrices.

From a biomedical standpoint, this radial gradient in mechanical performance may confer functional advantages for peripheral nerve conduits. The deformability moduli observed in the hydrogel conduit samples mirror the mechanical profile of native peripheral nerve tissue, where the outer layers—comparable to the epineurium—exhibited lower elasticity values, while the inner layers—resembling the perineurium—demonstrated higher elasticity values. A more flexible inner core could support axonal growth, while the stiffer outer layer offers protection against deformation from external mechanical stresses, compression, and shear forces encountered in the physiological environment. [27]. Native nerve tissue demonstrates higher moduli values on the perineurium and lower on the epineurium, as does the majority of the hydrogel conduits [28]. This alignment of deformability moduli between the hydrogel conduits and native nerve tissues demonstrates successful biomimicry of the hierarchical mechanical architecture of peripheral nerves.

Tensile strength is defined as the maximum uniaxial stress a hydrogel can withstand before failure or fracture occurs. It reflects the ultimate load-bearing capacity of the hydrogel under tension. Tensile strength provides insight into the hydrogel’s mechanical robustness and structural integrity under stretching forces. It is particularly important for scaffolds and implants that must resist tension in physiological conditions, ensuring mechanical compatibility and functional stability during application. An increase in tensile strength correlated positively with prolonged UV curing times, ranging from 10 to 45 min. This enhancement is attributed to increased crosslink density arising from extended photoactivation of methacrylate moieties, resulting in a more compact and robust polymeric network [29]. Samples exposed to 30–45 min of UV light (C- and D-series) exhibited significantly higher tensile strength compared to those cured for 10–20 min (A- and B-series).

Hydrogels plasticized with TEC, for example, A1–3 (TEC, 10 min UV) and B1–3 (TEC, 20 min UV), demonstrated superior tensile strength, especially at intermediate (1.5%) and higher (2.0%) concentrations. The relatively hydrophobic character of TEC fosters interchain hydrogen bonding and hydrophobic interactions, enhancing network compaction and limiting fluid uptake. This results in reduced hydrolytic degradation and improved mechanical resistance under tensile loading [30].

In contrast, glycerol-plasticized hydrogels, such as AA1–3 (glycerol, 10 min UV) and BB1–3 (glycerol, 20 min UV), exhibited lower tensile strengths, particularly at elevated concentrations. The hydrophilicity of glycerol promotes greater swelling through enhanced fluid retention, which increases polymer chain mobility and diminishes mechanical cohesion, thereby compromising tensile integrity.

A non-linear relationship between plasticizer concentration and tensile strength was observed. At low concentrations (1.0%), plasticizers slightly enhanced flexibility with marginal gains in tensile strength. Optimal tensile performance in TEC-containing samples was attained at 1.5%, balancing plasticization without excessive matrix softening. High plasticizer concentrations (2.0%) led to decreased mechanical properties in glycerol-containing hydrogels due to overhydration and matrix loosening. TEC samples at this concentration showed a plateau or slight decline, possibly due to phase separation or hindered crosslinking efficiency [31].

#### 2.2.3. Influence of Crosslinking Time on Swelling, Erosion, and Mechanical Properties

Crosslinking duration strongly impacted network density, thus influencing all key physicochemical parameters. Longer UV curing times (30–45 min) promoted increased vinyl group consumption (evidenced by heightened FTIR peak intensities at 1630–1640 cm^−1^), resulting in denser matrices with reduced swelling and enhanced tensile strength. For example, tensile strength peaked at 8.20 MPa in sample C1 (1.0% TEC, 30 min UV) and began to decline at 45 min, noted in the D-series (TEC, 45 min UV), likely due to UV overexposure leading to brittleness or polymer scission.

Erosion followed an inverse trend, where shorter UV exposure times (10–20 min) were associated with faster degradation. Samples cured for 45 min exhibited the lowest erosion rates, for example, D1 (1.0% TEC, 45 min UV) at 6.81 ± 3.60%, affirming the stabilizing effect of extended crosslinking. Glycerol-containing conduits did not benefit substantially from prolonged UV curing, underscoring the dominant role of plasticizer chemistry in network hydration.

Hydrogels containing TEC demonstrated a time-dependent relationship between swelling and matrix resilience. In samples cured for 10–20 min, increased swelling typically correlated with reduced MR, indicating a plasticization-dominated weakening of the matrix network. This effect was prominent in A1 and B1 on days 1–3, suggesting that insufficient curing may leave the network vulnerable to hydrolytic expansion. Conversely, in samples cured for 30–45 min, higher swelling was paradoxically associated with increased MR, as seen in samples C1 (1.0% TEC, 30 min UV), D1 (1.0% TEC, 45 min UV), and D3 (2.0% TEC, 45 min UV). This may reflect a more robust covalent network formation at extended curing times, where moderate swelling acts to redistribute stress without structural compromise, supported by the increase in intensity of the C=C stretching (1630 cm^−1^ to 1640 cm^−1^) peaks in C1 (1.0% TEC, 30 min UV), and D1 (1.0% TEC, 45 min UV), compared to A1 (1.0% TEC, 10 min UV) and B1 (1.0% TEC, 20 min UV) shown in Figure 2 [16]. Notably, D3 (2.0% TEC, 45 min UV) maintained both high swelling and high MR, indicating optimal crosslink-plasticizer synergy at 2.0% TEC with 45 min curing.

In glycerol-plasticized samples, UV curing did not prevent swelling-induced degradation due to persistent matrix loosening [24]. Glycerol-containing hydrogels exhibited inconsistent behavior at low concentrations (1.0% *v*/*v*), with samples such as AA1 (1.0% glycerol, 10 min UV) and BB1 (1.0% glycerol, 20 min UV) showing decoupled trends between swelling and MR. These irregularities may stem from glycerol’s hygroscopic nature, which could modulate fluid retention independently of network relaxation, introducing variability in mechanical response. Notably, when curing time increased to 30 min, depicted in CC1 (1.0% glycerol, 30 min UV), both swelling and MR stabilized, indicating a temporary window of structural equilibrium. However, this effect diminished beyond 30 min, with samples DD1–DD3 (glycerol, 45 min UV) demonstrating decreased MR in both regions despite stable or increased swelling.

Conduits subjected to shorter UV curing durations (e.g., 10 min) exhibited a notably higher deformability modulus (DM) in their inner regions compared to the corresponding outer matrix. This phenomenon can be attributed primarily to UV light attenuation, which results in radial gradients in crosslinking density across the hydrogel conduit. During photo-crosslinking, UV light initiates the polymerization of methacrylated groups primarily at the surface, with limited penetration into the hydrogel’s core. Consequently, the outer regions undergo a higher degree of covalent crosslinking, forming a stiffer, less extensible network.

Glycerol-containing samples were observed to have a lower modulus with increased curing time. This is likely due to the lower swelling observed in these samples, as a higher swelling results in a higher modulus from osmotic pressure stiffening the network [32,33]. Glycerol acts as a hygroscopic plasticizer, interrupting intermolecular hydrogen bonding, increasing free volume and swelling, and thus reducing network cohesion. However, in under-crosslinked systems, swelling-mediated osmotic stress paradoxically elevates modulus while increasing variability. This accounts for the modulus values of sample set A (glycerol, 10 min UV, and TEC 10 min UV), which had a wider variation [34]. Notably, samples C2 (1.5% TEC, 30 min UV) and C3 (2.0% TEC, 30 min UV) displayed a high modulus value on day 5 of 665.34 (±103.32) and 944.67 (±38.21), respectively. This corresponds to lower percentage swelling, suggesting a decrease in polymer chain mobility. TEC-containing samples had less variation after longer curing times and formed more stable networks within the GG system, resulting in smaller but more consistent modulus values. This is supported by lower erosion rates for samples containing TEC [35]. In contributing to bio-mimicry, the Youngs Modulus of porcine peripheral nerves were reported to be 181 kPa [36], suggesting samples BB1, BB2, and BB3 (glycerol, 20 min UV) and samples C1 and C3 (1.0% TEC, 30 min UV, and 2.0% TEC, 30 min UV) as ideal candidates for nerve conduits.

The high standard deviation observed in the mechanical properties can be attributed to the intrinsic hydrophilic nature of gellan gum hydrogels. These materials continuously absorb and release water in response to environmental conditions, leading to fluctuations in their internal water content. Since water uptake affects the polymer chain mobility, crosslink density, and overall network structure, even slight differences in swelling state between samples can significantly impact mechanical measurements. This dynamic equilibrium process is inherently variable and difficult to control, which explains the observed variability in results [32].

#### 2.2.4. The Contributing Effects of Each Crosslinking Approach

Each crosslinking stage contributed uniquely to the hydrogel’s final performance.

Thermal gelation created initial physical networks via helix formation, critical for initial shape retention. UV-induced covalent crosslinking added mechanical robustness and shape fidelity. It significantly reduced plasticizer mobility, especially in TEC-based samples, improving tensile strength and reducing matrix resilience loss. Ionic crosslinking with Mg^2+^ stabilized the network further by binding carboxylate moieties on GG, introducing reversible, secondary electrostatic bridges. FTIR evidence includes shifts in the C–O stretch (1000–1200 cm^−1^), confirming ionic interactions [16].

This tiered approach allowed precise control of internal gradients, aligning with the mechanical stratification observed in peripheral nerve fascicles. FTIR spectroscopy confirmed all critical chemical transitions. The C=C Stretch observed at 1630–1640 cm^−1^ increased intensity with UV exposure, confirming methacrylate polymerization. The C=O stretch at ~1720 cm^−1^ indicated ester formation, validating methacrylation and TEC plasticization. A C–O stretch found between 1045–1080 cm^−1^ confirmed glycerol incorporation, while intensity changes were observed with change in concentration. The C–H stretch (2850–2950 cm^−1^) was attributed to ethyl groups of TEC with a decline in intensity supporting crosslinking-induced immobilization [16].

These peaks support a molecular-level mechanism where triple-crosslinking modifies GG’s backbone to form a compact, elastic, and degradable scaffold. The increasing C=C peak intensity with UV time and the persistence of carboxylate-associated bands post-Mg^2+^ immersion provide direct chemical proof of successful, multi-step crosslinking with preserved hydration capacity.

#### 2.2.5. The Impact of Plasticizer Selection on Cell Viability

Sample C1 (1.0% TEC, 30 min UV) demonstrated superior cell growth compared to BB1 (1.0% glycerol, 20 min UV). This difference can be attributed to the distinct physicochemical properties of plasticizers. Glycerol, being highly hygroscopic, can create localized osmotic imbalances by drawing water away from cells, thereby generating a less favorable microenvironment for cellular attachment and proliferation. In contrast, triethyl citrate exhibits lower hydrophilicity and a more stable interaction with the polymer matrix, supporting a hydrated yet osmotically balanced surface that is more conducive to cell viability and growth [34].

## 3. Conclusions

This comprehensive analysis underscores the delicate balance required to optimize methacrylated GG hydrogels as a neuromaterial for peripheral nerve regeneration. Mechanical performance, particularly tensile strength, fracture energy, deformability moduli, and matrix resilience, was intricately tied to swelling and erosion behavior, both of which were strongly influenced by plasticizer type, concentration, and UV curing duration. TEC-based hydrogels consistently exhibited lower swelling and erosion, enhanced tensile strength, and superior mechanical integrity, particularly at 1.5–2.0% concentrations and with extended curing times (30–45 min). In contrast, glycerol-based hydrogels promoted swelling and elasticity but suffered from rapid erosion and reduced tensile properties at higher concentrations. Radial heterogeneity further altered the mechanical landscape, with inner matrix regions demonstrating greater resilience and fracture energy due to differential crosslinking during MgCl_2_ exposure. These gradients, while challenging, offer functional advantages by combining flexibility and structural support within a single construct. Ultimately, the interplay between crosslinking kinetics, plasticizer interactions, and hydration dynamics dictates hydrogel behavior, with TEC-based formulations showing the most promise for long-term nerve conduit applications. To ensure clinical relevance, future designs must carefully tailor crosslinking profiles and plasticizer systems to achieve a harmonized, anisotropic mechanical performance capable of mimicking native neural tissue layers and biomechanical environments.

## 4. Materials and Methods

### 4.1. Materials

Gellan gum (low acyl Gelzan™), triethyl citrate, glycerol, methacrylate anhydride, and phosphate-buffered saline (tablet, pH 7.2–7.6) were purchased from Sigma Aldrich. Paraffin liquid (uniLAB™) was purchased from Merck, Darmstadt, Germany. Magnesium chloride heptahydrate CP (MgCL_2_·7H_2_O) and acetone were purchased from Associated Chemical Enterprises, Southdale, Johannesburg, South Africa. Dulbecco’s Modified Eagle Medium (DMEM), Donor Equine Serum (DES), and Fetal Bovine Serum (FBS) were sourced from Hyclone (Separations, Johannesburg, South Africa). The Penicillin–Streptomycin–Amphotericin B (P/S/AB) solution was obtained from Lonza (Morristown, NJ, USA), while the Cell Proliferation Kit I was supplied by Roche and purchased through Sigma-Aldrich (St. Louis, MO, USA).

### 4.2. Synthesis of Hydrogel Conduits

**Figure 13:**
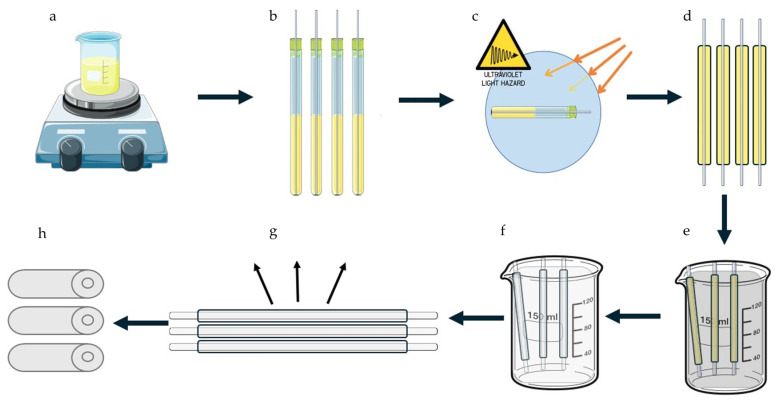
A graphical depiction of the formulation process. (**a**) The solvent was prepared and heated, then cooled before (**b**) being added to a lubricated mold. (**c**) The conduits were then cured under UV-A light, (**d**) then unmolded, (**e**) before being submerged first in cold acetone, then in (**f**) the MgCl_2_ bath. (**g**) Conduits were then dried, before the inner portion of the mold was removed to produce (**h**) the final product.

Hollow nerve conduits were prepared using the solvent casting technique with dimensions comparable to the rat model of sciatic nerve repair. A GG-based hydrogel was produced by dissolving low-acyl GG in deionized water at a concentration of 1.5% *w*/*v* and heating the resultant solution to between 85 °C and 90 °C. The solution was maintained at this temperature for 30 min. Predetermined amounts of triethyl citrate and glycerol were added for their plasticizing properties. Methacrylate anhydride was added at a concentration of 1% *v*/*v* as a photo initiator. The hydrogel solution was poured into a cylindrical mold lubricated with liquid paraffin and cured under UV-A light (320 to 400 nm wavelength) for predetermined time intervals. The resultant conduit was dried under laminar flow for 24 h before being soaked in cold acetone for 1 h to remove excess methacrylate anhydride, then dried under laminar flow overnight to evaporate any excess acetone. Finally, the conduit was cross-linked in 150.00 mL of a 50% *w*/*v* MgCl_2_ solution for 12 h, followed by a last drying step for a further 12 h to remove any access solvent. Figure 13 below describes the formulation process.

**Figure 14:**
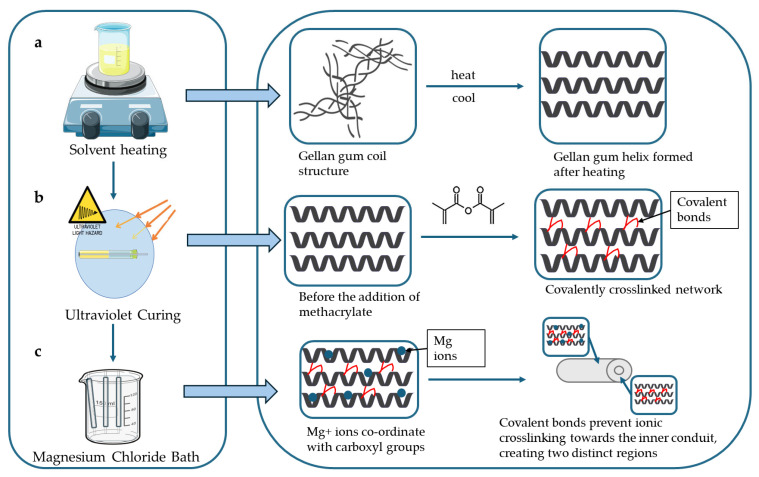
A graphical depiction of crosslinking mechanisms involved in the hydrogel conduit synthesis. It describes the (**a**) coil to helix transition, (**b**) the covalent crosslinking using methacrylate anhydride, and (**c**) ionic crosslinking involving carboxyl groups.

The formulation process involved three distinct crosslinking mechanisms, depicted in Figure 14. Initially, heating the Gellan Gum solution to 90 °C followed by cooling induced thermotropic gelation, resulting in a structural transition from random coil to double helix (a). Subsequently, covalent crosslinking was achieved through UV-initiated polymerization of methacrylate groups, forming stable covalent bonds between Gellan Gum chains (b). Finally, immersion of the conduits in an MgCl_2_ solution facilitated ionic crosslinking by coordinating divalent magnesium ions with the negatively charged carboxylate groups along the polymer backbone (c) [37].

Several conduits were formulated using different plasticizer ratios in order to achieve optimal mechanical performance. Table 1 below shows the difference in composition between each sample. Briefly, conduits varied in triethyl citrate (TEC) concentration (1.0–2.0% *v*/*v*), glycerol concentration (1.0–2.0% *v*/*v*), and curing time (10–45 min). Controls describe conduits made from GG only, from GG and plasticizer only with no UV crosslinker, and GG with methacrylate anhydride (MAA) and no plasticizer, cured for 20 min.

### 4.3. Determination of Fluid-Uptake and Swelling Ratio

Swelling ratio is defined as the mass (or volume) of fluid absorbed by the hydrogel relative to its dry mass (or volume) at equilibrium.

Wherein *Ws* refers to the swollen weight and *Wi* refers to the initial weight. Samples of 15 mm conduits weighing between 100–250 mg (n = 3) were emersed in PBS at pH 7.4 and maintained at a temperature of 37 °C in order to simulate in vivo conditions. Samples were removed at designated timepoints and weighed to assess swelling using the following equation:

(1) $$Swelling\;ratio\;=\;\frac{Ws-Wi}{Wi}\;\times 100$$

### 4.4. Erosion Studies

Erosion refers to the mass loss of the hydrogel over time due to enzymatic, hydrolytic, or mechanical degradation. It is quantified using the following equation:

(2) $$\%\;ER\;=\;\frac{Wt-W0}{W0}\;\times 100$$

wherein *W*0 refers to the initial dry weight and *Wt* refers to the dry weight of a sample after a specified time. Samples were maintained in the same conditions described in swelling studies and removed at predetermined intervals (n = 3). Samples were then dried under laminar flow for 24 h before the eroded weight was taken.

### 4.5. Determination of Compressive Textural Profiling

Textural analysis of the hydrogel conduits was determined using a Texture Analyser (TAXTplus Stable Microsystems, Surrey, UK) fitted with a 10 mm diameter Delrin probe, undertaken on hydrated conduit samples at pre-determined sampling points (n = 3). The Matrix Resilience (MR), Deformability Modulus (DM), and Fracture Energy (FE) were calculated using the resulting textural profiles.

MR (N.sec) was calculated using Force-Time profiles, as the ratio of compression to decompression of the area under the curve (AUC). The resultant values indicated the ability of the conduit to store and release mechanical energy without undergoing permanent deformation. DM (N/mm) indicates the conduit’s ability to undergo elastic deformation and describes the flexibility and stiffness of the hydrogel conduit. This was determined using the resultant Force–Distance profiles, measured from the initial point (zero) to the maximum recorded force.

FE (N.mm) was determined from the Force–Distance profiles as well, represented by the AUC, and is defined as the critical energy required to propagate a crack through the hydrogel matrix.

The parameters used include a pre-test and post-test speed of 1.00 mm/s, a test speed of 0.5 mm/s, a trigger force of 0.5 N, and a load cell of 5 kg with a target mode of 10% strain [38].

### 4.6. Tensile Strength and Young’s Modulus Determination

Determination of the tensile strength of the hydrated conduits (n = 3) at pre-determined sampling points was conducted using a Texture Analyser (TAXT.plus Stable Microsystems, Surrey, UK) fitted with articulated tensile grips and a 5 kg load cell.

A 20 mm length of conduit was fixed at a distance of 10 mm between the two clamps with a test speed of 0.5 mm/s and a 0.5 N trigger force. The target mode was a distance of 20 mm.

Force (N) at the breaking point of the Force–Time profiles was measured. In order to determine tensile strength (MPa), conduit dimensions were measured using a digital Vernier caliper (Krafft, DV150GW, Schoellerstr Düren, Germany), and the cross-sectional area was determined. The following equation was used to calculate tensile strength [39]:

(3) $$Tensile\;Stregnth\;(MPa)\;=\;\frac{Force\;at\;breaking\;point\;(N)}{Cross\;sectional\;area\;of\;sample\;(mm^{2})}$$

Young’s Modulus was determined from the above test, calculated as the slope of the stress vs. strain graph.

### 4.7. FTIR Analysis

Samples were analyzed over an FTIR spectra of wavelengths between 4000–650 cm^−1^ (20 scans per spectra) at a resolution of 4 cm^−1^ and a constant pressure of 110 psi, using a PerkinElmer Spectrum 2000 ATR-FTIR (PerkinElmer 100, Llantrisant, Wales, UK) spectrometer fitted with a single reflection diamond MIRTGS detector.

### 4.8. In Vitro Cell Proliferation Assessment

The rat adrenal gland pheochromocytoma PC12 mixed adherent/suspension cell line (PC-12) obtained from Cellonex, Separations, South Africa, was cultured in a 5% CO_2_ atmosphere at 37 ◦C. Culture media was replaced every 2 days and consisted of DMEM supplemented with 10% *v*/*v* DES, 5% *v*/*v* FBS, and 1% *v*/*v* P/S/AB solution. Cell proliferation on conduits containing different plasticizers was assessed using the MTT proliferation assay with UV-absorbance measured at 570 nm. Samples of 5 mm × 5 mm were sterilized under UVC light for 30 min [38] then incubated overnight in 200 µL of culture medium in a 96-well plate. These samples were then seeded with PC12 cells at a density of 1 × 10^4^ cells per well and incubated for 24 and 48 h. The positive control consisted of wells containing only cells and culture medium, while the negative control comprised wells treated with DMSO. Studies were performed in triplicate (n = 3).

### 4.9. Statistical Analysis

All data were expressed as mean values ± standard deviation.

## Figures and Tables

**Table 1:** Description of different conduits and their composition.

Sample Description	Curing Time (Minutes)	Triethyl Citrate Concentration (*v*/*v*)	Glycerol Concentration (*v*/*v*)
A1	10	1.0	0
A2	10	1.5	0
A3	10	2.0	0
AA1	10	0	1.0
AA2	10	0	1.5
AA3	10	0	2.0
B1	20	1.0	0
B2	20	1.5	0
B3	20	2.0	0
BB1	20	0	1.0
BB2	20	0	1.5
BB3	20	0	2.0
C1	30	1.0	0
C2	30	1.5	0
C3	30	2.0	0
CC1	30	0	1.0
CC2	30	0	1.5
CC3	30	0	2.0
D1	45	1.0	0
D2	45	1.5	0
D3	45	2.0	0
DD1	45	0	1.0
DD2	45	0	1.5
DD3	45	0	2.0
GELLAN GUM	0	0	0
TRIETHYL CITRATE	0	1.5	0
GLYCEROL	0	0	1.5
METHACRYLATE	20	0	0

## Data Availability

The original contributions presented in this study are included in the article. Further inquiries can be directed to the corresponding author.

## Abbreviations

The following abbreviations are used in this manuscript:

GG	Gellan Gum
MAA	Methacrylic acid
PNI	Peripheral nerve injury
NGC	Nerve guidance conduit
FTIR	Fourier transform infrared spectroscopy
PBS	Phosphate-buffered saline
MR	Matrix Resilience
DM	Deformability Modulus
FE	Fracture Energy
TS	Tensile Strength
AUC	Area under the curve
